# Localization of Components of the RNA-Degrading Machine in *Bacillus subtilis*

**DOI:** 10.3389/fmicb.2016.01492

**Published:** 2016-09-21

**Authors:** Nora Cascante-Estepa, Katrin Gunka, Jörg Stülke

**Affiliations:** Department of General Microbiology, Institute of Microbiology and Genetics, Georg-August-Universität GöttingenGöttingen, Germany

**Keywords:** protein localization, microbial cell biology, RNA degradation, RNase Y, RNA degradosome

## Abstract

In bacteria, the control of mRNA stability is crucial to allow rapid adaptation to changing conditions. In most bacteria, RNA degradation is catalyzed by the RNA degradosome, a protein complex composed of endo- and exoribonucleases, RNA helicases, and accessory proteins. In the Gram-positive model organism *Bacillus subtilis*, the existence of a RNA degradosome assembled around the membrane-bound endoribonuclease RNase Y has been proposed. Here, we have studied the intracellular localization of the protein that have been implicated in the potential *B. subtilis* RNA degradosome, i.e., polynucleotide phosphorylase, the exoribonucleases J1 and J2, the DEAD-box RNA helicase CshA, and the glycolytic enzymes enolase and phosphofructokinase. Our data suggests that the bulk of these enzymes is located in the cytoplasm. The RNases J1 and J2 as well as the RNA helicase CshA were mainly localized in the peripheral regions of the cell where also the bulk of messenger RNA is localized. We were able to demonstrate active exclusion of these proteins from the transcribing nucleoid. Taken together, our findings suggest that the interactions of the enzymes involved in RNA degradation in *B. subtilis* are rather transient.

## Introduction

The survival of an organism depends on its ability to rapidly adapt to changes of the environmental conditions. This ability arises from the control of gene expression, which allows a different set of proteins to be available for each new condition. While this regulation can occur at different levels, the protein quantity ultimately depends on the availability of mRNA. The amounts of these RNAs are determined by the rates of transcription and degradation (RNA turnover). Moreover, the stability and functionality of many mRNAs rely on post-transcriptional events which are referred to as RNA processing, and which are essential in all domains of life. However, the regulation of the mRNA stability is particularly important in bacteria as these organisms have a short lifespan, and need to adapt immediately to harsh changes and stresses in their environment. Indeed, the bacterial mRNAs have a half-life of only few minutes (Hui et al., 2014), which allows the bacteria to respond swiftly to any challenge.

The enzymes directly responsible for the degradation of the mRNAs are the ribonucleases (RNases), as they catalyze the exo- or endoribonucleolytic cleavage of a phosphodiester bond from an RNA molecule. The RNases interact and collaborate with several other enzymes that act on RNA to ensure an accurate regulation of mRNA stability and, thus, of gene expression. These auxiliary enzymes include RNA-helicases, poly(A) polymerases, pyrophosphohydrolases, and oligoribonucleases (Bechhofer, 2011; Laalami et al., 2014; Mohanty and Kushner, 2016). The RNases and RNA-related proteins often interact within the cell to form complexes, the RNA-degrading machines, probably to enhance the activity and/or specificity of the individual enzymes. In Archaea and Eukarya, the conserved exosome is the most versatile RNA-degrading machine. Interestingly, the ring-like structure of the exosome resembles the structural organization of the bacterial polynucleotide phosphorylase (PNPase; Evguenieva-Hackenberg et al., 2014; Kilchert et al., 2016). In bacteria, a structurally different complex, the RNA degradosome, has been detected (Py et al., 1994). Although this type of complex has been found in several Gram-positive and Gram-negative species as well as in chloroplasts, these RNA-degrading complexes are best studied in the model organisms *Escherichia coli* and *Bacillus subtilis* (Lee and Cohen, 2003; Carpousis, 2007; Kaberdin and Lin-Chao, 2009; Górna et al., 2012; Lehnik-Habrink et al., 2012; Ait-Bara and Carpousis, 2015; Giraud et al., 2015). In *E. coli*, the RNA degradosome consists of the essential endoribonuclease RNase E, the exoribonuclease PNPase, the RNA helicase RhlB, and the glycolytic enzyme enolase (Eno; Carpousis, 2007). The latter is important for the degradation of the glucose transporter mRNA during phosphosugar stress (Morita et al., 2004). The RNA degradosome forms around the central RNase E, whose intrinsically unstructured C-terminal tail serves as a scaffold for the interacting proteins. Moreover, RNase E contains a membrane-associated domain that guides the whole complex to the cytoplasmic membrane. This membrane association was recently confirmed by fluorescence microscopy studies (Khemici et al., 2008; Strahl et al., 2015).

In *B. subtilis*, an RNA degradosome has been proposed based on co-purification and binary protein–protein interaction experiments. This potential complex is thought to be a functional analog to the *E. coli* degradosome, although several key proteins are not conserved at the sequence level. A central endoribonuclease, RNase Y, serves as the scaffold of the complex, interacting with the exoribonucleases PNPase and RNase J1, the DEAD-box RNA-helicase CshA and two glycolytic enzymes, Eno and phosphofructokinase (PfkA; Commichau et al., 2009; Lehnik-Habrink et al., 2010; Newman et al., 2012; Salvo et al., 2016). The role of the glycolytic enzymes in the *B. subtilis* degradosome has not yet been elucidated. Furthermore, RNase J1 also interacts with its paralog, RNase J2 (Commichau et al., 2009; Mathy et al., 2010). RNase Y harbors a transmembrane domain at its N-terminus and localizes to the membrane (Lehnik-Habrink et al., 2011; Bührmann et al., 2012). Even though the interactions between these proteins have been detected by a variety of methods in different labs, the proposed *B. subtilis* RNA degradosome has, unfortunately, never been purified as a whole complex. Moreover, the localization of all the components of the RNA-degrading complex has not been studied yet at the single-cell level.

Here we have investigated the subcellular localization of the proteins involved in RNA degradation in *B. subtilis*. To address this question, we have performed fluorescence microscopy experiments with live cells expressing green fluorescent protein (GFP) fusions of the respective components of the putative *B. subtilis* RNA degradosome. We confirmed the membrane localization of the RNase Y. However, no other protein studied under these conditions was found to be exclusively membrane-associated. Interestingly, we observed RNases J1 and J2, as well as the DEAD-box RNA-helicase CshA, to have a ribosome-like distribution, indicating that they co-localize with the bulk of mRNA in the cell.

## Materials and Methods

### Bacterial Strains and Growth Conditions

The *B. subtilis* strains used in this study are listed in **Table 1**. *E. coli* DH5α (Sambrook et al., 1989) was used for plasmid constructions and transformation using standard techniques (Sambrook et al., 1989). Luria Bertani (LB) broth was used to grow *E. coli* and *B. subtilis*. When required, media were supplemented with antibiotics at the following concentrations: ampicillin 100 μg ml^-1^ (for *E. coli*) and kanamycin (10 μg ml^-1^), chloramphenicol (5 μg ml^-1^), or spectinomycin 150 μg ml^-1^ (for *B. subtilis*). Sporulation (SP) plates were prepared by the addition of 15 g l^-1^ Bacto agar (Difco) to SP medium (8 g of nutrient broth per liter, 1 mM MgSO_4_, 13 mM KCl, supplemented after sterilization with 2.5 μM FeSO_4_, 500 μM CaCl_2_, and 10 μM MnCl_2_). *B. subtilis* was transformed with plasmid DNA according to the two-step protocol (Kunst and Rapoport, 1995). Transformants were selected on SP plates containing antibiotics as above.

**Table 1:** *Bacillus subtilis* strains used in this study.

Strain	Genotype	Source^a^
168	*trpC2*	Laboratory collection
CCB434	W168 *ΔrnjA::spec*	Figaro et al. (2013)
GP594	*trpC2 Δeno::cat*	Commichau et al. (2013)
GP1035	*trpC2*Δ*cshA::aphA3*	Lehnik-Habrink et al. (2013)
GP1048	*trpC2 rnjA-Strep-cat rnjB-3xFLAG-spec*	Lehnik-Habrink (2011)
GP1684	*trpC2 amyE::(Pxyl-rny-mSFgfpV206K spec)*	pGP1449 → 168
GP1694	*trpC2 amyE::(Pxyl-mSFgfpV206K-rnjA spec)*	pGP2802 → 168
GP1695	*trpC2 amyE::(Pxyl-mSFgfpV206K-rnjB spec)*	pGP2803 → 168
GP1698	*trpC2 pnpA-gfpA206K spec*	pGP2806 → 168
GP1699	*trpC2 rnjB-gfpA206K spec*	pGP2807 → 168
GP1700	*trpC2 eno-gfpA206K spec*	pGP2808 → 168
GP1720	*trpC2 pfkA-gfpA206K spec*	pGP2810 → 168
GP1721	*trpC2 cshA-gfpA206K spec*	pGP2811 → 168
GP1722	*trpC2 rnjA-gfpA206K spec*	pGP2812 → 168
GP1747	*trpC2*Δ*pfkA::aphA3*	See Materials and Methods
GP1748	*trpC2*Δ*pnpA::aphA3*	See Materials and Methods
GP2502	*trpC2*Δ*rnjA::spec*	CCB434 → 168

^a^*Arrows indicate construction by transformation*.

For the fluorescence microscopy imaging, *B. subtilis* cultures in exponential phase (OD_600_ 0.2–0.6) or stationary phase (OD_600_ 1.5) were incubated for 5 min at 37°C and 200 rpm with DAPI and Nile Red at a final concentration of 1 μg ml^-1^, to stain the bacterial chromosome and the cell membrane, respectively. To interrupt transcription in the cells, cultures were incubated for 10 min at 37°C and 200 rpm with rifampicin (dissolved in methanol) at a final concentration of 200 μg ml^-1^. As a negative control, the same amount of methanol was added to the cells. The expression of GFP fusions under the control of a xylose-inducible promoter was induced by addition of 0.1% xylose.

### Plasmid Constructions

All plasmids used in this study are listed in **Table 2**. To express *B. subtilis* proteins under the control of their native expression signals, fused to the monomeric GFP, we constructed plasmid pBP43. For this purpose, we amplified the *gfp* gene fragment using the primer pair ML220/ML221 (Rothe et al., 2013) and plasmid pSH3 as the template (Oliva et al., 2010). The PCR product was digested with *Hin*dIII and cloned into the vector pUS19 (Benson and Haldenwang, 1993). The vector pBP43 allows the construction of GFP fusions and integration of the plasmid into the chromosome via Campbell-type recombination between the cloned gene fragment and the chromosomal copy of the gene^1^. For the fusion of proteins implicated in RNA degradation to the GFP, we amplified the 3′ 600 bp (lacking a stop codon) of each gene, with the respective oligonucleotide pair (see Supplementary Table S1), using chromosomal DNA of *B. subtilis* 168 as the template. The PCR products were cloned between the *Bam*HI and *Sal*I sites of pBP43. The resulting plasmids were pGP2806, pGP2807, pGP2808, pGP2810, pGP2811, pGP2812 for *pnpA*, *rnjB*, *eno*, *pfkA*, *cshA*, and *rnjA*, respectively. Integration of these plasmids into the chromosome of *B. subtilis* leads to the in-frame fusion of the *gfp* alleles to the entire genes lacking their stop codon.

**Table 2:** Plasmids used in this study.

Plasmid	Relevant characteristics	Primers	Reference
pBP43	pUS19-*gfpA206K*	ML220/ML221	This study
pDG780	Amplification of the *aphA3* cassette		Guérout-Fleury et al. (1995)
pGP1449	pHJS105-*rny*	NC51/NC52NC53/NC54	This study
pGP2802	pHJS105-*rnjA*	NC63/NC64	This study
pGP2803	pHJS105-*rnjB*	NC65/NC2	This study
pGP2806	pBP43-*pnpA*	ML91/ML92	This study
pGP2807	pBP43-*rnjB*	ML173/ML174	This study
pGP2808	pBP43-*eno*	FR86/FR90	This study
pGP2810	pBP43-*pfkA*	ML56/ML57	This study
pGP2811	pBP43-*cshA*	ML11/ML12	This study
pGP2812	pBP43-*rnjA*	ML58/ML59	This study
pHJS105	*amyE::(Pxyl-mSFgfpV206K spec)*	–	Gamba et al. (2015)
pSH3	*Pxyl-divIVA-gfpA206K*	–	Oliva et al. (2010)
pUS19	pUC19-*spec*	–	Benson and Haldenwang (1993)

For the ectopic inducible overexpression of N-terminal GFP fusions, we used plasmid pHJS105 that allows integration into the *amyE* locus and induction of the fusion by the addition of xylose (Jahn et al., 2015). For the construction of the plasmids we amplified the *rnjA* and *rnjB* genes from chromosomal DNA of *B. subtilis* 168 using the oligonucleotide pairs NC63/NC64 and NC65/NC2, respectively. We then cloned the PCR fragments between the *Bam*HI and *Hin*dIII sites of pHJS105 to obtain plasmids pGP2802 and pGP2803, respectively. RNase Y encoded by the *rny* gene has a N-terminal transmembrane helix (Lehnik-Habrink et al., 2011). Therefore, we had to fuse the GFP to the C terminus of RNase Y. For this purpose, we amplified the *rny* gene from chromosomal DNA of *B. subtilis* 168 and the *gfp* gene from pHJS105 using the primer pairs NC51/NC52 and NC53/NC54, respectively. Both PCR fragments were digested with *Bam*HI and fused together. Subsequently, we cloned the resulting fragment between the *Avr*II and *Not*I sites of pHJS105. The resulting plasmid, pGP1449, allows the overexpression of RNase Y with a C-terminal GFP upon induction with xylose.

### Construction of Deletion Strains

Deletion of the *pfkA* and *pnpA* genes was achieved by transformation with PCR products constructed using oligonucleotides (see Supplementary Table S1) to amplify DNA fragments flanking the target genes and intervening antibiotic resistance cassettes as described previously (Guérout-Fleury et al., 1995; Wach, 1996).

### Microscopy

For fluorescence microscopy, cells were grown at 28°C in LB medium to an OD_600_ of 0.2–0.5 (unless otherwise indicated). Fluorescence images were obtained with the AxioImager M2 fluorescence microscope, equipped with digital camera AxioCam MRm and AxioVision Rel 4.8 software for image processing and an EC Plan-NEOFLUAR 100X/1.3 objective (Carl Zeiss, Göttingen, Germany). The applied filter sets were the Filter set 49 (G 365, FT 395, BP 445/50; Carl Zeiss) for DAPI detection, the Filter set 38 (BP 470/40, FT 495, BP 525/50; Carl Zeiss) for GFP detection, and Filter set 43 (BP 545/25, FT 570, BP 605/70) for Nile Red visualization. The overlays of fluorescent and phase-contrast images were prepared for presentation with ImageJ 1.49p (National Institutes of Health, Bethesda, USA). Images were taken with an exposure time of 1 s for the GFP constructs (except for Eno-GFP, exposure time 500 ms), 500 ms for the membrane stain Nile Red, and 100 ms for the DNA stain DAPI.

### Detection of the RNase J1–J2 Complex

To study the formation of the complex formed between the RNases J1 and J2, we used strain *B. subtilis* GP1048 which encodes RNase J1 fused to a Strep-tag to facilitate purification *via* a StrepTactin column (IBA GmbH, Göttingen, Germany) and RNase J2 fused to a triple FLAG tag to allow immunological detection. The bacteria were cultivated and Step-tagged protein purified as described previously (Meyer et al., 2011). The identity of the proteins was verified by Western blot analysis using antibodies directed against the Strep and the FLAG-tag, respectively.

## Results

### Construction and Functional Characterization of GFP Fusions to Proteins Implicated in RNA Degradation

Several RNases, RNA helicases, and glycolytic enzymes were proposed to form an RNA-degrading complex in *B. subtilis*. However, the subcellular localization of most of these proteins has not yet been determined by *in vivo* fluorescence labeling. The possibility of fusing fluorescent proteins to visualize the *in vivo* localization of proteins has greatly improved our understanding of the cell dynamics in the past years. However, the ability of the GFP to dimerize can cause artifacts in the localization of the fusion protein within the cells, which can be avoided by the use of monomeric fluorescent proteins (Miyawaki, 2011; Margolin, 2012). Moreover, the addition of a tag to a protein could affect its functionality and localization *in vivo*, by avoiding interactions, conformational changes, or proper folding (Gunka et al., 2013). Finally, overexpression of labeled proteins may result in aggregation and mislocalization (Miyawaki, 2011; Margolin, 2012). To study the localization of the proteins implicated in RNA degradation in *B. subtilis*, we therefore used a monomeric GFP variant (Oliva et al., 2010) and integrated the constructs into the chromosome, ensuring that the labeled protein is expressed from its native promoter. Additionally, we verified that these fusion proteins had retained their native activity *in vivo*.

**FIGURE 1:**
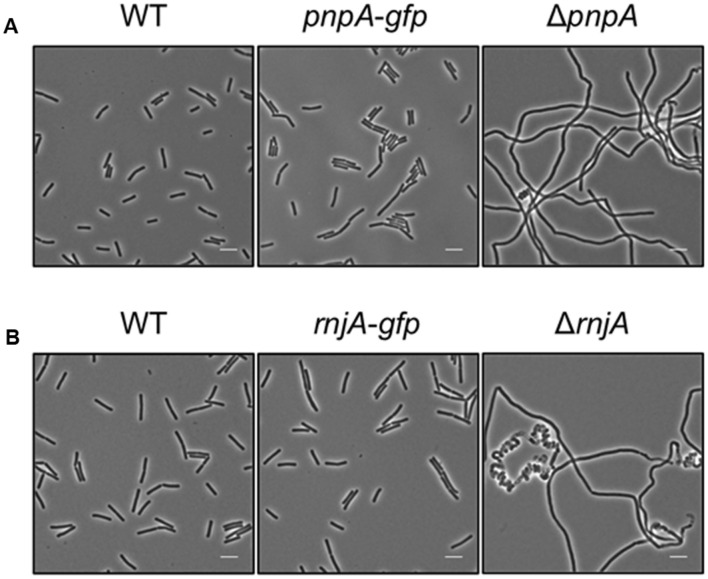
**The strains harboring the GFP fusions to PnpA and RNase J1 show a wild type phenotype**. Light microscopy of strains **(A)** 168 (WT), GP1698 (*pnpA-gfp*), and GP1748 (Δ*pnpA*) and **(B)** 168 (WT), GP1722 (*rnjA-gfp*), and GP2502 (Δ*rnjA*). The strains were grown in LB medium at 37°C to stationary phase. Both strains harboring the GFP fusions form short cells comparable to the wild type strain and are not elongated as their respective deletion mutants. Scale bar, 5 μm. WT, wild type.

**FIGURE 2:**
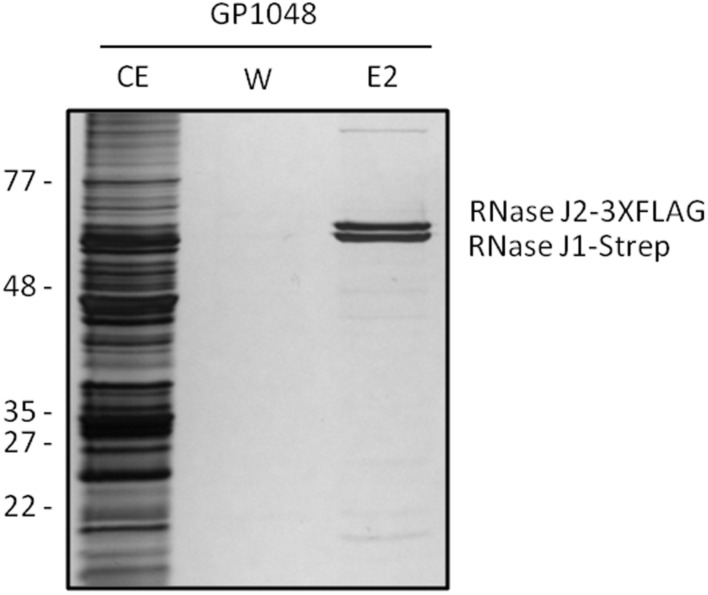
**RNases J1 and J2 form a complex *in vivo***. *Bacillus subtilis* GP1048 was cultivated in LB medium at 37°C to OD_600_ of 1.0. Cells were disrupted and Strep-RNase J1 was purified by a StrepTactin column. CE, cell extract; W, wash fraction; E2, elution fraction 2. The identity of RNases J1 and J2 was proven by Western blot analysis (data not shown). The faint eluted bands at the top and the bottom of the gel correspond to PycA and AccB, respectively, the two biotin-containing proteins of *B. subtilis* (Meyer et al., 2011).

**FIGURE 3:**
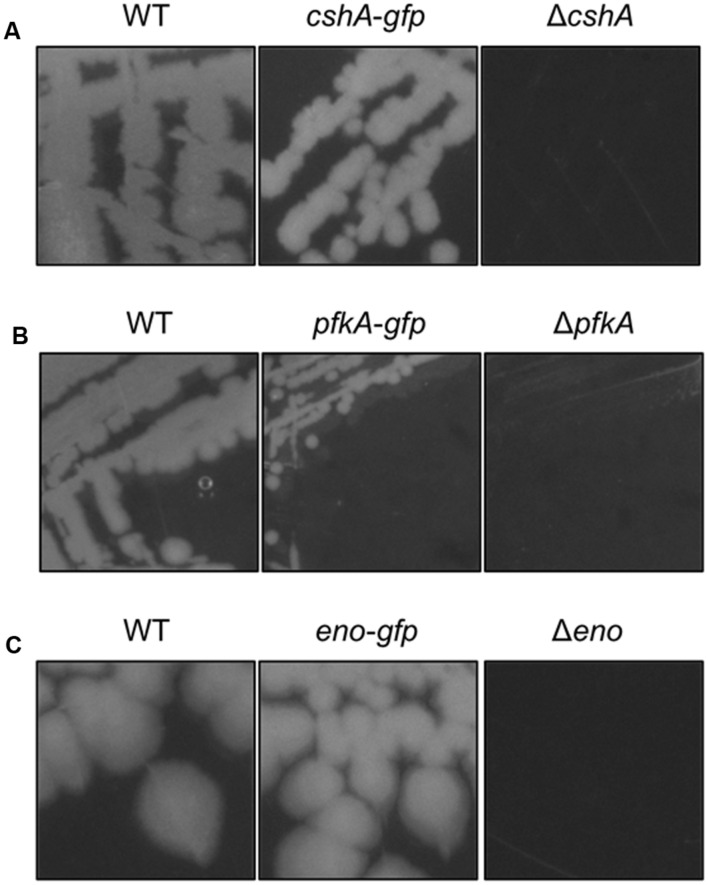
**The strains harboring the GFP fusions to CshA, PfkA, and Enolase do not show growth defects**. **(A)** Strains 168 (WT), GP1721 (*cshA-gfp*), and GP1035 (Δ*cshA*) were streaked on LB agar and incubated at 28°C overnight. The strain harboring the *cshA-gfp* fusion in the genome is able to grow under these conditions while the deletion mutant has a growth defect. **(B)** Strains 168 (WT), GP1720 (*pfkA-gfp*), and GP1747 (Δ*pfkA*) were streaked on CE minimal medium plates with 0.5% glucose and incubated for 48 h at 37°C. The strain with the *pfkA-gfp* fusion is able to grow under these conditions, in contrast to the deletion mutant. **(C)** Strains 168 (WT), GP1700 (*eno-gfp*), and GP594 (Δ*eno*) were streaked on LB plates and incubated at 37°C overnight. The strain harboring the *eno-gfp* fusion can grow under these conditions, while the deletion mutant is unable to grow.

Based on the rationale that if the fusions rendered the proteins inactive the strains would show a phenotype similar to the respective deletion mutants, we decided to compare the reporter strains to the respective wild type and deletion mutant strains. The deletion of the *pnpA* gene encoding PNPase causes the cells to grow in long cell chains and results in cold sensitivity (Wang and Bechhofer, 1996). *B. subtilis* GP1698 harboring the PnpA-GFP fusion was able to grow with a normal cell length (**Figure 1A**). RNase J1, encoded by the *rnjA* gene, has been regarded as essential until recently. Although the construction of a deletion mutant is possible, this strain shows severely affected cell morphology, with cells growing in curly long chains (Figaro et al., 2013). Our strain GP1722 (RNase J1-GFP) showed normal cell morphology and no chains (**Figure 1B**). Although RNase J2, the paralog of RNase J1, can be deleted without any phenotype, it has been shown to interact *in vitro* with RNase J1 with a 1:1 stoichiometry (Mathy et al., 2010). Moreover, we have observed a strong interaction of both proteins by *in vivo* co-purification (see **Figure 2**). As there is no assay for the functionality of RNase J2, we would take a similar localization of RNases J1 and J2 as an indication that the GFP tag did not affect J2 localization (see below). RNase Y is encoded by *rny*, the first gene of the bicistronic *rny-ymdB* operon (Diethmaier et al., 2011). To avoid interference with the expression of the phosphodiesterase YmdB, we constructed fusion expressing RNase Y-GFP under the control of a xylose-inducible promoter. As membrane localization of RNase Y has been demonstrated in several independent studies (Hunt et al., 2006; Lehnik-Habrink et al., 2011; Bührmann et al., 2012), no further functional analyses were performed. The *cshA* deletion mutant shows poor growth at temperatures lower than 28°C (Lehnik-Habrink et al., 2013) but strain GP1721 was able to grow at 28°C like the wild type (**Figure 3A**). The essentiality of the glycolytic enzymes Eno and PfkA has recently been revisited (Commichau et al., 2013). Eno was shown to be essential for the growth of *B. subtilis* in LB medium, while the *pfkA* gene is not essential but is required for growth on minimal medium with glucose as the carbon source. For strain GP1720 expressing the PfkA fused to GFP, we observed growth on minimal medium with glucose as the carbon source, indicating activity of the fusion protein (**Figure 3B**). The strain encoding the fusion of Eno to GFP (GP1700) was viable and grew as the wild type strain indicating functionality of Eno when fused to GFP (**Figure 3C**).

### Localization of Proteins Implicated in RNA Degradation and Turnover

**FIGURE 4:**
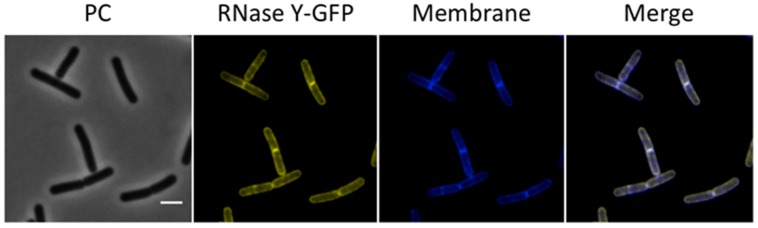
**Membrane localization of RNase Y**. Fluorescence microscopy of the strain GP1684 (Pxyl-*rny-gfp*) grown in LB medium at 28°C to stationary phase. The expression of the fusion protein was induced by addition of 0.1% xylose to the culture medium. The membrane was stained with Nile Red. RNase Y-GFP co-localizes with the Nile Red staining, confirming the membrane localization of the protein. Scale bar, 2 μm. PC, phase contrast.

RNase Y has been proposed to provide the scaffold for RNA-degrading enzymes in *B. subtilis*. Several studies have reported membrane localization of this protein (Hunt et al., 2006; Zweers et al., 2009; Lehnik-Habrink et al., 2011; Bührmann et al., 2012), and our results are in excellent agreement with those observations (**Figure 4**). Interestingly, we observed a higher intensity of RNase Y localization at the division septa. In a recent study, RNase Y was found to interact with the dynamin protein DynA, which also localizes to the division septa (Bührmann et al., 2012).

**FIGURE 5:**
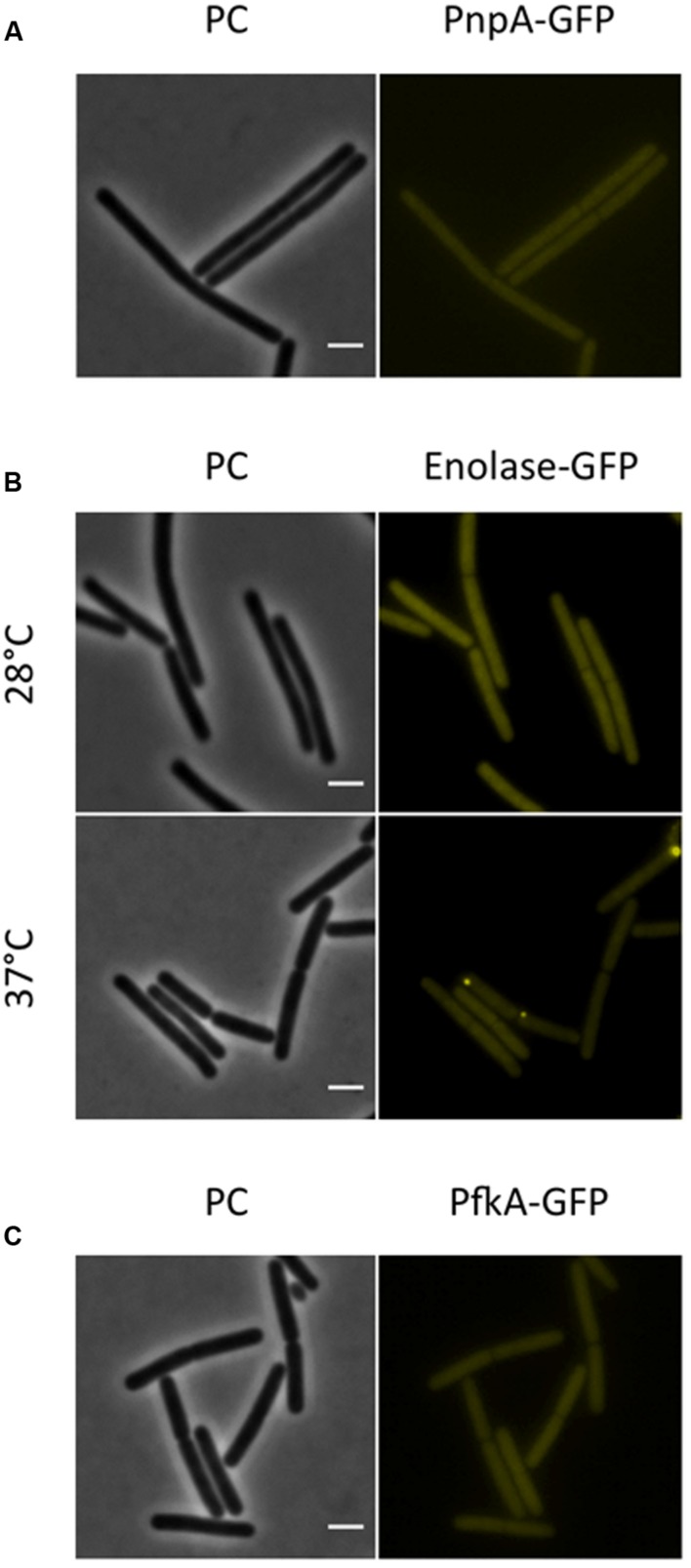
**The RNA-degrading exoribonuclease PnpA, as well as the glycolytic enzymes enolase and PfkA are localized to the cytoplasm, where they appear uniformly distributed**. Fluorescence microscopy of strains harboring GFP fusions. **(A)** The strains GP1698 (*pnpA-gfp*) was grown in LB medium at 28°C to mid-exponential phase. The GFP fusion protein can is localized to the cytoplasm, where it appears homogeneously distributed. **(B)** Strain GP1700 (*eno-gfp*) was grown to mid-exponential phase in LB medium at 28°C or 37°C. The protein appears widespread in the cytoplasm at both temperatures but at 37°C some cells show bright spots localized at the poles. **(C)** The strain GP1720 (*pfkA-gfp*) was grown in LB medium at 28°C to mid-exponential phase and the fusion protein is evenly distributed within the cytoplasm. Scale bar, 2 μm. PC, phase contrast.

**FIGURE 6:**
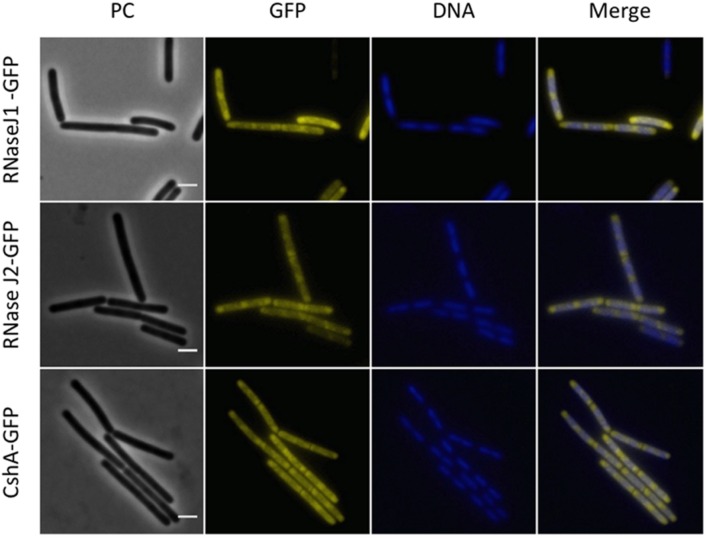
**Polar localization of the RNases J1 and J2 and the DEAD-box RNA helicase, CshA**. Fluorescence microscopy of strains GP1694 (*Pxyl-rnjA-gfp*), GP1695 (*Pxyl-rnjB-gfp*), and GP1721 (*cshA-gfp*). The cells were grown in LB medium at 28°C to mid-exponential phase. The expression of RNase J1-GFP and RNase J2-GFP was induced by addition of 0.1% xylose to the culture medium. The nucleoid was stained by DAPI as described in the Material and Methods section. The proteins are distributed in the cytoplasm concentrating at the poles. The concentration of the proteins is lower at the center of each cell, where the nucleoid is positioned as can be observed by DAPI stain. Scale bar, 2 μm. PC, phase contrast.

**FIGURE 7:**
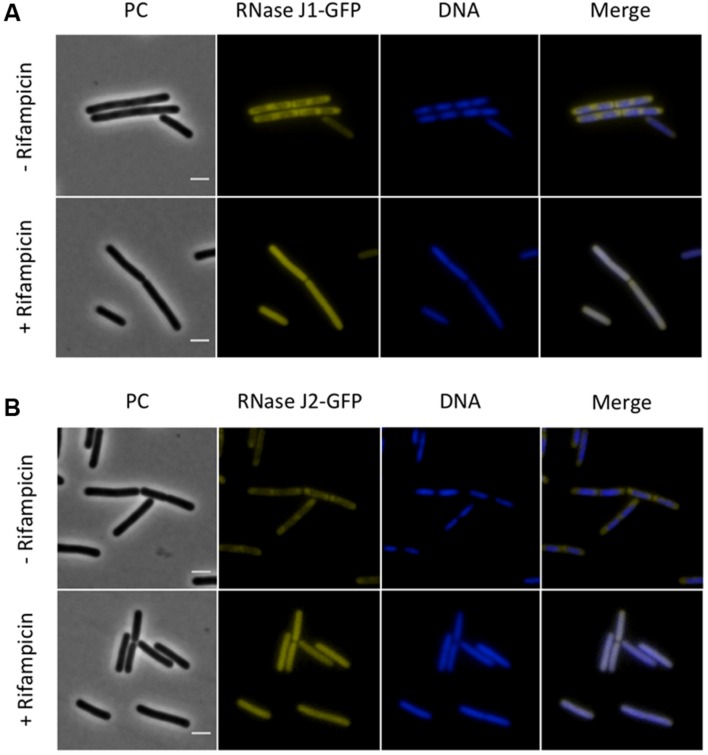
**The sub-polar localization of RNases J1 and J2 is lost upon inhibition of RNA synthesis by rifampicin**. Fluorescence microscopy of strains **(A)** GP1694 (*Pxyl-rnjA-gfp*) and **(B)** GP1695 (*Pxyl-rnjB-gfp*). Bacteria were grown in LB medium at 37°C to mid-exponential phase, when rifampicin or methanol were added to the cultures as described above. The expression of RNase J1-GFP and RNase J2-GFP was induced by addition of 0.1% xylose to the culture medium. The nucleoid was stained by DAPI as described in the Material and Methods section. The sub-polar localization of both RNases J1 and J2 is lost as the RNA synthesis is inhibited and the proteins appear evenly distributed in the cytoplasm. Upon addition of rifampicin the nucleoid, stained by DAPI, spreads occupying the majority of the cell volume. Scale bar, 2 μm. PC, phase contrast.

Next, we investigated the localization of the exoribonucleases PnpA, RNases J1 and J2. For PnpA, we observed a cytoplasmic localization with an even distribution throughout the cell (see **Figure 5A**). RNases J1 and J2 were found to localize in the cytoplasm except in the central region of the cell. A DAPI stain identified this latter region as the nucleoid, the RNases J1 and J2 are therefore localized in the cytoplasm around the nucleoid (**Figure 6**). In order to confirm the exclusion of RNases J1 and J2 from the nucleoid region, we treated cultures expressing the fusion proteins with rifampicin to stop transcription. This transcription stop results in relaxation of the nucleoid; as a result the nucleoid delocalizes from the central region of the cell to the whole cytoplasm (Hunt et al., 2006). This delocalization of the nucleoid is shown in **Figure 7**. It is accompanied by a complete delocalization of RNases J1 and J2, which now appeared evenly distributed throughout the cytoplasm (see **Figure 7**). Similar localization and delocalization pattern upon rifampicin have been shown previously for ribosomes in *B. subtilis* (Mascarenhas et al., 2001; Lehnik-Habrink et al., 2013). Taken together, these observations suggest that both RNases J1 and J2 are localized to those areas in the cytoplasm where the RNA target molecules are present. Interestingly, very similar results have been obtained for the localization of the RNase Y-associated DEAD box helicase, CshA (Hunger et al., 2006), and we were able to confirm the localization of CshA in the cytoplasmic area that does also harbor the ribosomes (Mascarenhas et al., 2001; see **Figure 6**). Interestingly, for *E. coli* RNase E delocalization has been observed in response to rifampicin treatment (Strahl et al., 2015); such a delocalization was not detected for RNase Y in *B. subtilis* (data not shown). This difference may be caused by the different attachment of the two RNases to the membrane: RNase E is membrane-attached *via* an amphipathic helix (Khemici et al., 2008), whereas RNase Y is inserted to the membrane *via* a trans-membrane helix (Lehnik-Habrink et al., 2011).

For the glycolytic enzymes Eno and PfkA, we detected an even distribution in the cytoplasm (see **Figure 5**). Interestingly, Eno formed bright spots at the polar regions of some cells at 37°C but not at 28°C. Polar localization of Eno has been reported before and was shown to depend on phosphorylation of the enzyme on a tyrosine residue by the protein tyrosine kinase PtkA (Jers et al., 2010).

## Discussion

In this work, we provide the first detailed analysis of localization of the proteins involved in RNA degradation in *B. subtilis*. Our results are somewhat in contradiction to the idea of an RNA degradosome in this bacterium: While RNase Y is located at the cytoplasmic membrane, the exoribonucleases RNase J1 and J2 as well as the RNA helicase CshA are localized in the peripheral regions of the cell, i.e., the region where the bulk of the RNA is localized. Moreover, the PNPase and the glycolytic enzymes are found homogeneously in the cytoplasm. It is tempting to speculate that the differential localization of PNPase as compared to the other exoribonucleases J1 and J2 and the helicase CshA is related to the fact, that PnpA also exerts functions that are not related to RNA degradation: PnpA does also bind single-stranded DNA and participates in DNA repair (Cardenas et al., 2009).

At a glance, these observations contradict previous reports on the interactions between the proteins involved in RNA degradation. However, there are several independent *in vivo* and *in vitro* reports that confirm the binary interactions of the potential RNA degradosome enzymes strongly suggesting that these interactions are real (Commichau et al., 2009; Lehnik-Habrink et al., 2010, 2011; Newman et al., 2012; Salvo et al., 2016). Prior attempts to localize the RNA degradosome proteins indicated that most of them can be found both at the membrane and in the cytoplasm, without allowing a decision about the preferred localization (Lehnik-Habrink et al., 2011). All potential degradosome proteins are likely to be very abundant proteins as has been shown for PnpA, PfkA, or Eno (Eymann et al., 2004). This hypothesis is supported by the fact that the genes encoding the degradosome proteins all belong to the most strongly expressed genes in *B. subtilis* (Nicolas et al., 2012). Thus, these abundant proteins may be present at multiple places in the cell, and they may serve multiple functions. Indeed, it has been shown that PNPase and the RNA helicase RhlB of *E. coli* are part of two distinct complexes: the two proteins form a complex in the cytoplasm, and they are also part of the membrane-attached RNA degradosome (Lin and Lin-Chao, 2005). Similarly, Eno is not only part of the degradosome but also a cytoplasmic glycolytic enzyme. Thus, it is important to study the relative localization of the proteins in the different compartments. It is interesting to note that in *E. coli* the major fraction of the RNA helicase RhlB localizes to the membrane via RNase E, even though the protein is also engaged in the cytoplasmic complex with PnpA (Lin and Lin-Chao, 2005; Strahl et al., 2015). In *B. subtilis*, the major fractions of the exoribonucleases and the RNA helicase CshA are clearly not associated to the membrane, i.e., these major fractions do not interact with RNase Y. Given the clear evidence for the multiple binary protein–protein interactions on one hand and the lack of purification of the *B. subtilis* RNA degradosome on the other, it is tempting to speculate that these interactions are rather transient, and may not always involve all components. Possibly, the RNA degrading enzymes engage in a variety of different interactions to achieve specificity in RNA degradation and processing. Indeed, it has recently been shown that the glycolytic enzyme glyceraldehyde 3-phosphate dehydrogenase (GapA) form a complex with RNase Y and RNase J1 to modulate J1 activity. This study also revealed that only a minor fraction of GapA (1–2%) interacts with RNase J1 and RNase Y (Gimpel and Brantl, 2016). Interestingly, this interaction of a minor part of the total GapA population in the cell correlates perfectly with the fact that GapA is a cytoplasmic protein, even though a part of the protein is found associated to the membrane (Meile et al., 2006; Hahne et al., 2008).

Taken together, RNA degradation in *B. subtilis* seems to be a highly dynamic process that involves several RNases and accessory proteins that interact with the RNase as required. It is tempting to speculate that additional factors that modulate RNase activities in *B. subtilis* are to be uncovered.

## Author Contributions

JS and NC-E designed the study and wrote the manuscript. NC-E and KG performed the experiments.

## Conflict of Interest Statement

The authors declare that the research was conducted in the absence of any commercial or financial relationships that could be construed as a potential conflict of interest. The reviewer KP and handling Editor declared their shared affiliation, and the handling Editor states that the process nevertheless met the standards of a fair and objective review.

## Footnotes

1 http://subtiwiki.uni-goettingen.de/wiki/index.php/PBP43
