# Supplementary material for: Localization of Components of the RNA-Degrading Machine in Bacillus subtilis
Source: Front Microbiol. 2016 Sep 21;7:1492. doi: 10.3389/fmicb.2016.01492 (PMC5030255; doi:10.3389/fmicb.2016.01492)
Supplement: Supplementary file 1 [file Table_1.DOCX]

TABLE S1. Oligonucleotides used in this study.

| **Primer** | **Sequence ^a^** | **Purpose** |
| --- | --- | --- |
| FR86 | TTTGTCGACCTTGTTTAAGTTGTAGAAAGAGTTGATACCGTGG | Rev; Amplification of *eno* (no STOP codon) for cloning into pBP43 |
| FR90 | AAAGGATCCCGCTTCAAACAATCGTTGAAGC | Fwd; Amplification of *eno* for cloning into pBP43 |
| ML11 | ACAGTCGACGTAAGATTTTTTCTGGCGTCTGTCACCTG | Rev; Amplification of *cshA* (no STOP codon) for cloning into pBP43 |
| ML12 | ACAGGATCCCTGGCTGAAGCTCTGAACCTTCG | Fwd; Amplification of *cshA* for cloning into pBP43 |
| ML56 | ACAGTCGACGATAGACAGTTCTTTTGAAAGCTGATACA | Rev; Amplification of p*fkA* (no STOP codon) for cloning into pBP43 |
| ML57 | ACAGGATCCGTGGAGACGGTTCCTATATGG | Fwd; Amplification of *pfkA* for cloning into pBP43 |
| ML58 | ACAGTCGACAACCTCCATAATGATCGGCAGG | Rev; Amplification of *rnjA* (no STOP codon) for cloning into pBP43 |
| ML59 | ACAGGATCCCGAACAATCAACCAGCTGTATCG | Fwd; Amplification of *rnjA* for cloning into pBP43 |
| ML91 | ACAGTCGACAGATTGTTGTTCTTCTTTTTCTTTCTCTTCAC | Rev; Amplification of *pnpA* (no STOP codon) for cloning into pBP43 |
| ML92 | ACAGGATCCGCTCGGAGATATGGACTTTAAAG | Fwd; Amplification of *pnpA* for cloning into pBP43 |
| ML173 | ACAGTCGACTACTTCCATAATAATTGGGATGATCATCG | Rev; Amplification of *rnjB* (no STOP codon) for cloning into pBP43 |
| ML174 | ACAGGATCCCTCATTTACTCTAAAACAGTAGATCTTC | Fwd; Amplification of *rnjB* for cloning into pBP43 |
| ML220 | ATAAAGCTTATGAGTAAAGGAGAAGAACTTTTCACTG | Fwd; Amplification of *gfp* from pSH3 |
| ML221 | ATAAAGCTTTTATTTGTATAGTTCATCCATGCCATGTG | Rev; Amplification of *gfp* from pSH3 |
| NC2 | TTTAAGCTTTTATACTTCCATAATAATTGGGATGATCATCGGTTTAC | Rev; Amplification of *rnjB* for cloning into pHJS105 |
| NC51 | AAACCTAGGATGACCCCAATTATGATGGTTCTCA | Fwd; Amplification of *rny* for cloning into pHJS105 |
| NC52 | TTTGGATCCTTTTGCATACTCTACGGCTCGA | Rev; Amplification of *rny* (no STOP codon) for cloning into pHJS105 |
| NC53 | AAAGGATCCGGCTCAGGAAGCGGTATGGGTACCCTGCAGATGAG | Fwd; Amplification of *gfp* from pHJS105 |
| NC54 | TTTGCGGCCGCTTATTTGTAGAGCTCATCCATGCCA | Rev; Amplification of *gfp* from pHJS105 (with STOP codon) |
| NC63 | AAAGGATCCAAATGAAATTTGTAAAAAATGATCAGACTG | Fwd; Amplification of *rnjA* for cloning into pHJS105 |
| NC64 | TTTAAGCTTTTAAACCTCCATAATGATCGGCAG | Rev; Amplification of *rnjA* for cloning into pHJS105 |
| NC65 | AAAGGATCCAAATGAAAAAGAAAAATACAGAAAACGTTAG | Fed; Amplification of *rnjB* for cloning into pHJS105 |
| NC107 | AATTTGAAGCCGTGGGACCG | Fwd; Amplification of upstream fragment (deletion of *pfkA*) |
| NC108 | *CCGGTGATATTCTCATTTTAGCC*ATTCTCCATTCACCTCAGCAACATA | Rev; Amplification of upstream fragment (deletion of *pfkA*) |
| NC109 | *TATTATATTTTACTGGATGAATTGTTTTAG*TGTACAGCTGAAGGCTGAAGAT | Fwd; Amplification of downstream fragment (deletion of *pfkA*) |
| NC110 | GCATGATCGCATCTGTTCCG | Rev; Amplification of downstream fragment (deletion of *pfkA*) |
| NC111 | ATGGCTAAAATGAGAATATCACCGGA | Fwd; Amplification of *aphA3* (from pDG780) |
| NC112 | CTAAAACAATTCATCCAGTAAAATATAATA | Rev; Amplification of *aphA3* (from pDG780) |
| NC113 | ATCTGGCCGATGTGGATTGC | Fwd; Sequencing in *aphA3* |
| NC114 | AGATGTTGCTGTCTCCCAGG | Rev; Sequencing in *aphA3* |
| NC115 | TCCGATATGGATCTAAGTGCGG | Fwd; Sequencing *pfkA* deletion |
| NC116 | TGCATTGTTTGAACTGCTTCAACC | Rev; Sequencing *pfkA* deletion |
| NC126 | *CCGGTGATATTCTCATTTTAGCCAT*ACAATTACGAACTCCTCTCTTCG | Rev; Amplification of upstream fragment (deletion of *pnpA*) |
| NC127 | ATCGGGTTTCCGACAGCGA | Fwd; Amplification of upstream fragment (deletion of *pnpA*) |
| NC128 | *TATTATATTTTACTGGATGAATTGTTTTAG*ATGAAAACATAAAAGGAGCCTGGG | Fwd; Amplification of downstream fragment (deletion of *pnpA*) |
| NC129 | CCATTATGTATCTTGCTTAACACTC | Rev; Amplification of downstream fragment (deletion of *pnpA*) |
| NC130 | ATTAAAGGAATTGTCATTCATGGTG | Fwd; Sequencing *pnpA* deletion |
| NC131 | GTTCAGTGACTGTTCCAAGCG | Rev; Sequencing *pnpA* deletion |

**^a^** Underlined bases indicate restriction sites. *Italic* indicates *aphA3^R^*.
